# Supplementary material for: Computational Modeling to Identify Drugs Targeting Metastatic Castration-Resistant Prostate Cancer Characterized by Heightened Glycolysis
Source: Pharmaceuticals (Basel). 2024 Apr 29;17(5):569. doi: 10.3390/ph17050569 (PMC11124089; doi:10.3390/ph17050569)
Supplement: Supplementary file 1 [file pharmaceuticals-17-00569-s001.zip › Supplementary Figures.pdf]

Supplemental Materials

Computational modeling to identify drugs targeting metastatic castration-resistant prostate cancer characterized by heightened glycolysis

Authors: Mei-Chi Su[1], Adam M. Lee[1], Weijie Zhang[2], Danielle Maeser[2], Robert F. Gruener[1], Yibin Deng[3], R. Stephanie Huang[1,2]*

Affiliations:

1. Department of Experimental and Clinical Pharmacology, College of Pharmacy, University of Minnesota, Minneapolis, MN 55455, USA
2. Bioinformatics and Computational Biology, University of Minnesota, Minneapolis, MN 55455, USA
3. Department of Urology, Masonic Cancer Center, University of Minnesota Medical School, Minneapolis, Minnesota, USA

Addresses for Correspondence:
R. Stephanie Huang, PhD
Experimental and Clinical Pharmacology
University of Minnesota College of Pharmacy
B-138 Phillips-Wangensteen Building
Minneapolis, MN 55455, United States
Phone: 612-625-1372
Email: rshuang@umn.edu

Sources of Funding:

RSH received funding from NIH/NCI Grants R01CA204856, R01CA229618 and NCI Contract No. 75N91019D00024, Task Order No. 75N91020F00003. She also received funding from the University of Minnesota (UMN) OACA Faculty Research Development grant, a GIA award, a SURRGE award from the College of Pharmacy and a Masonic Cancer Center CRTI Exceptional Translational Research award.

Disclosures: The authors have no other relevant affiliations or financial involvement with any organization or entity with a financial interest in or financial conflict with the subject matter or materials discussed in the manuscript.

Conflict of Interest Statement: The authors declare no conflicts of interest.

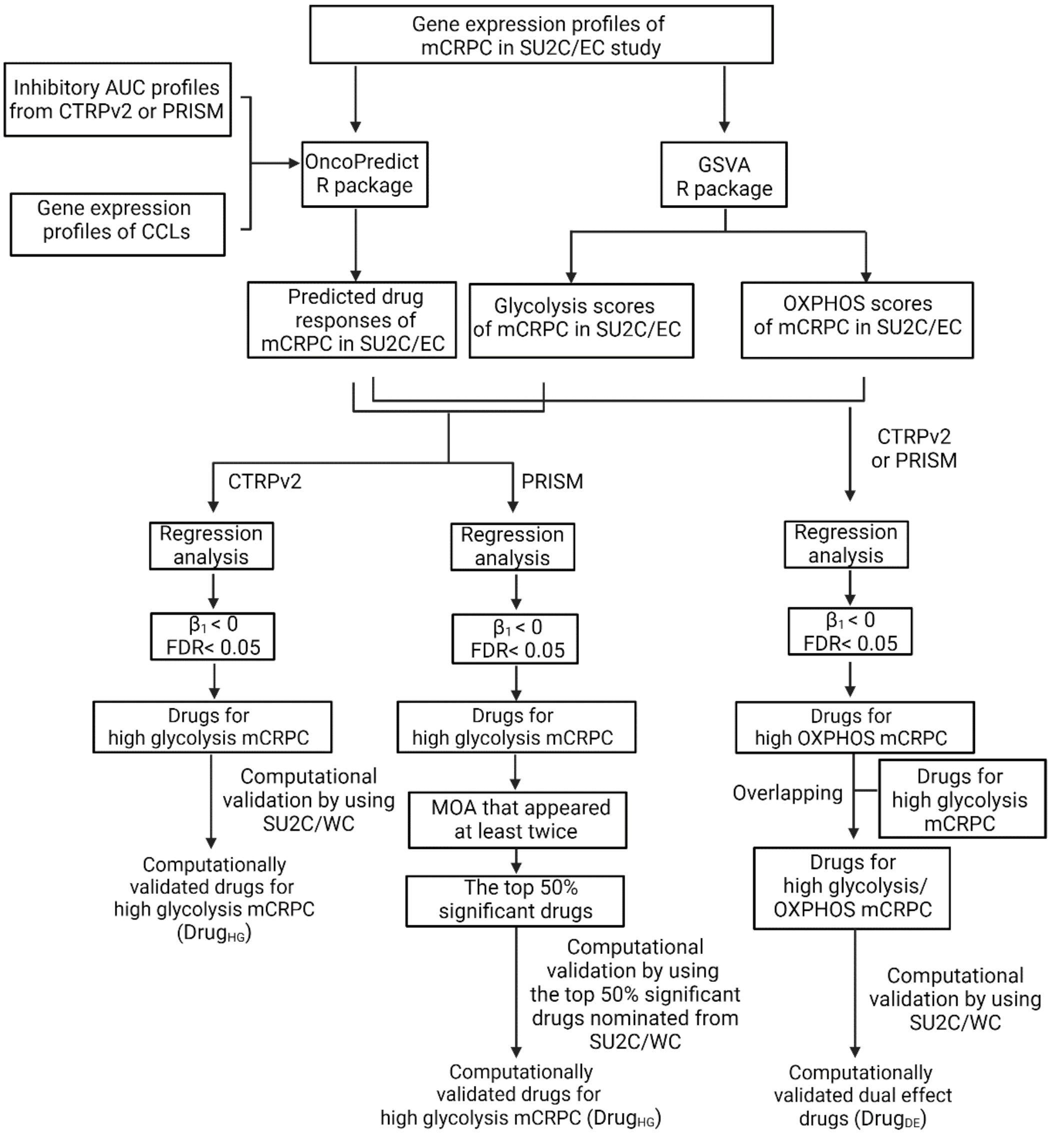

Figure S1. Block diagram of drug candidate nominations.

The nomination of drug candidates began by exploring the relationship between gene expression levels and drug responses using datasets from in vitro cancer cell lines, specifically CTRPv2 and PRISM. This relationship was then leveraged to estimate drug responses in patients with mCRPC based on the gene expression profiles of their tumors. Regression analysis was used to identify potential drug candidates based on the correlation coefficients between predicted drug responses and the glycolysis and OXPHOS scores as well as corresponding statistical significance. Additionally, an independent clinical study, SU2C/WC, was used for validation, enhancing the robustness of selected drug candidates. Abbreviations: CTRPv2: Cancer Therapeutics Response Portal Version 2; PRISM: Profiling Relative Inhibition Simultaneously in Mixtures; CCLs: Cancer Cell Lines; SU2C/EC: Standard Up to Cancer East Coast; SU2C/WC: Standard Up to Cancer West Coast; GSVA: Gene Set Variation Analysis; β_1: a coefficient of predicted drug response of mCRPC tumors; FDR: false discovery rate; mCRPC: metastatic castration-resistant prostate cancer; OXPHOS: oxidative phosphorylation; MOA: mechanism of action.

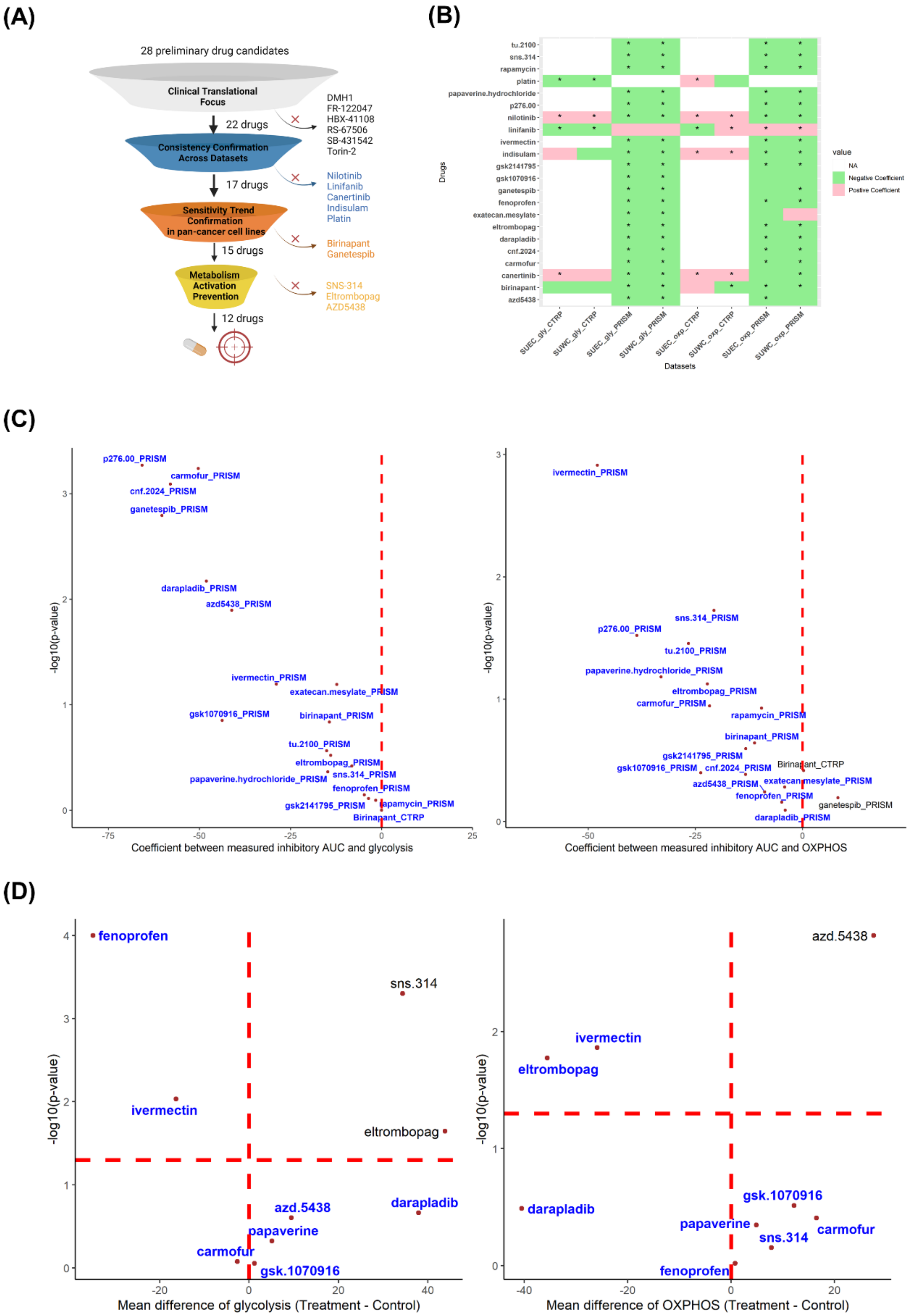

(A)
28 preliminary drug candidates
Clinical Translational Focus
DMH1
FR-122047
HBX-41108
RS-67506
SB-431542
Torin-2
22 drugs
Consistency Confirmation Across Datasets
Nilotinib
Linifanib
Canertinib
Indisulam
Platin
17 drugs
Sensitivity Trend Confirmation in pan-cancer cell lines
Birinapant
Ganetespib
15 drugs
Metabolism Activation Prevention
SNS-314
Eltrombopag
AZD5438
12 drugs
(B)
tu.2100
sns.314
rapamycin
platin
papaverine.hydrochloride
p276.00
nilotinib
linifanib
ivermectin
indisulam
gsk2141795
gsk1070916
ganetespib
fenoprofen
exatecan.mesylate
eltrombopag
darapladib
cnf.2024
carmofur
canertinib
birinapant
azd5438
Drugs
value
NA
Negative Coefficient
Postive Coefficient
SUEC_gly_CTRP
SUWC_gly_CTRP
SUEC_gly_PRISM
SUWC_gly_PRISM
SUEC_oxp_CTRP
SUWC_oxp_CTRP
SUEC_oxp_PRISM
SUWC_oxp_PRISM
Datasets
(C)
-log10(p-value)
Coefficient between measured inhibitory AUC and glycolysis
p276.00_PRISM
carmofur_PRISM
cnf.2024_PRISM
ganetespib_PRISM
darapladib_PRISM
azd5438_PRISM
ivermectin_PRISM
exatecan.mesylate_PRISM
gsk1070916_PRISM
birinapant_PRISM
tu.2100_PRISM
eltrombopag_PRISM
sns.314_PRISM
papaverine.hydrochloride_PRISM
fenoprofen_PRISM
gsk2141795_PRISM
rapamycin_PRISM
Birinapant_CTRP
Coefficient between measured inhibitory AUC and OXPHOS
ivermectin_PRISM
sns.314_PRISM
p276.00_PRISM
tu.2100_PRISM
papaverine.hydrochloride_PRISM
eltrombopag_PRISM
carmofur_PRISM
rapamycin_PRISM
birinapant_PRISM
gsk2141795_PRISM
Birinapant_CTRP
gsk1070916_PRISM
cnf.2024_PRISM
azd5438_PRISM
exatecan.mesylate_PRISM
fenoprofen_PRISM
ganetespib_PRISM
darapladib_PRISM
(D)
-log10(p-value)
Mean difference of glycolysis (Treatment - Control)
fenoprofen
sns.314
ivermectin
eltrombopag
darapladib
azd.5438
papaverine
carmofur
gsk.1070916
Mean difference of OXPHOS (Treatment - Control)
azd.5438
ivermectin
eltrombopag
gsk.1070916
darapladib
papaverine
carmofur
sns.314
fenoprofen

Figure S2. Step-by-step results of the four-step filtering procedure. (A) The flow chart of the number of drug candidates selected after each screening step (B) Heatmap of the coefficient of relationship between imputed drug responses and glycolysis/OXPHOS score across different datasets (CTRPv2 and PRISM). The statistical analysis was performed by Student's t-test. *: FDR <0.05 (C) Dot plot of coefficients between measured inhibitory AUC and glycolysis (left) or OXPHOS (right) in pan-cancer CCLs. The red dashed vertical line indicates a coefficient equal to 0. The statistical analysis was performed by Student's t-test (D) Dot plot of the change of glycolysis (left) and OXPHOS (right) after 10 μM drug treatment for 24 hours in PC3 cells. The red dashed horizontal line indicates a p-value equal to 0.05 and the red dashed vertical line indicates a mean difference equal to 0. The statistical analysis was performed by Welch's t-test. Of note, the perturbation-driven gene expression profiles of CNF2024, Exatecan mesylate, GSK2141795, P276-00, Rapamycin, and TU.2100 are not available in CMAP. Abbreviations: SUEC: Standard Up to Cancer East Coast; SUWC: Standard Up to Cancer East Coast; gly: glycolysis; oxp: oxidative phosphorylation; FDR: false discovery rate.

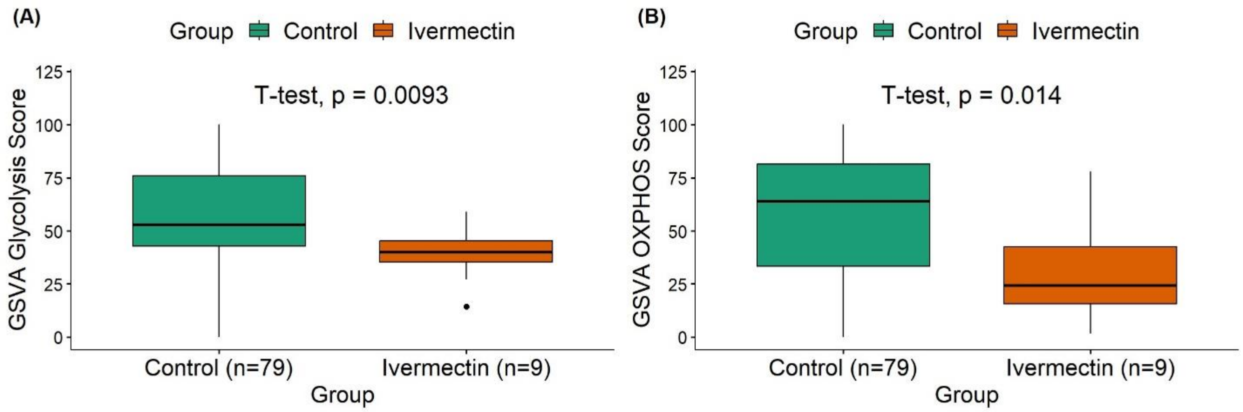

Figure S3. Alternations in cancer metabolism activity after ivermectin treatment. (A) change of glycolysis score after 24 hours of ivermectin treatment in PC3 cells (B) change of OXPHOS score after 24 hours of ivermectin treatment in PC3 cells. The statistical analysis was performed by Welch's t-test. Data sourced from CMAP. Abbreviations: GSVA: Gene set variation analysis; OXPHOS: oxidative phosphorylation.

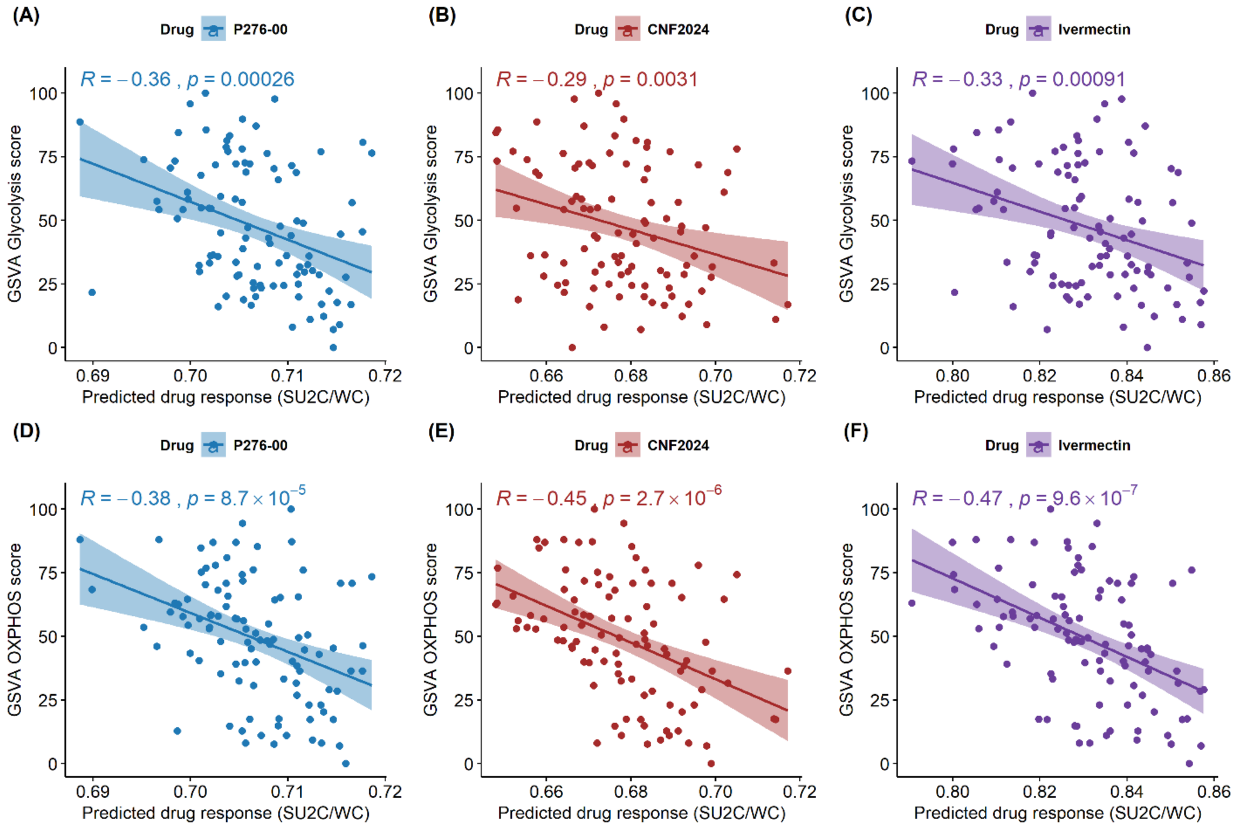

Figure S4. The correlation between glycolysis scores or OXPHOS scores with the predicted drug response in mCRPC patients in SU2C/WC clinical studies. (A-C) Correlations between the glycolysis score and the predicted patient response to P276-00, CNF2024, and ivermectin, respectively (D-F) Correlations between the OXPHOS score and the predicted patient response to P276-00, CNF2024, and ivermectin, respectively. For each correlation plot, the Spearman correlation coefficient and its p-value are given. The statistical analysis was performed by Spearman rank correlation and Student's t-test with a significance level α=0.05.

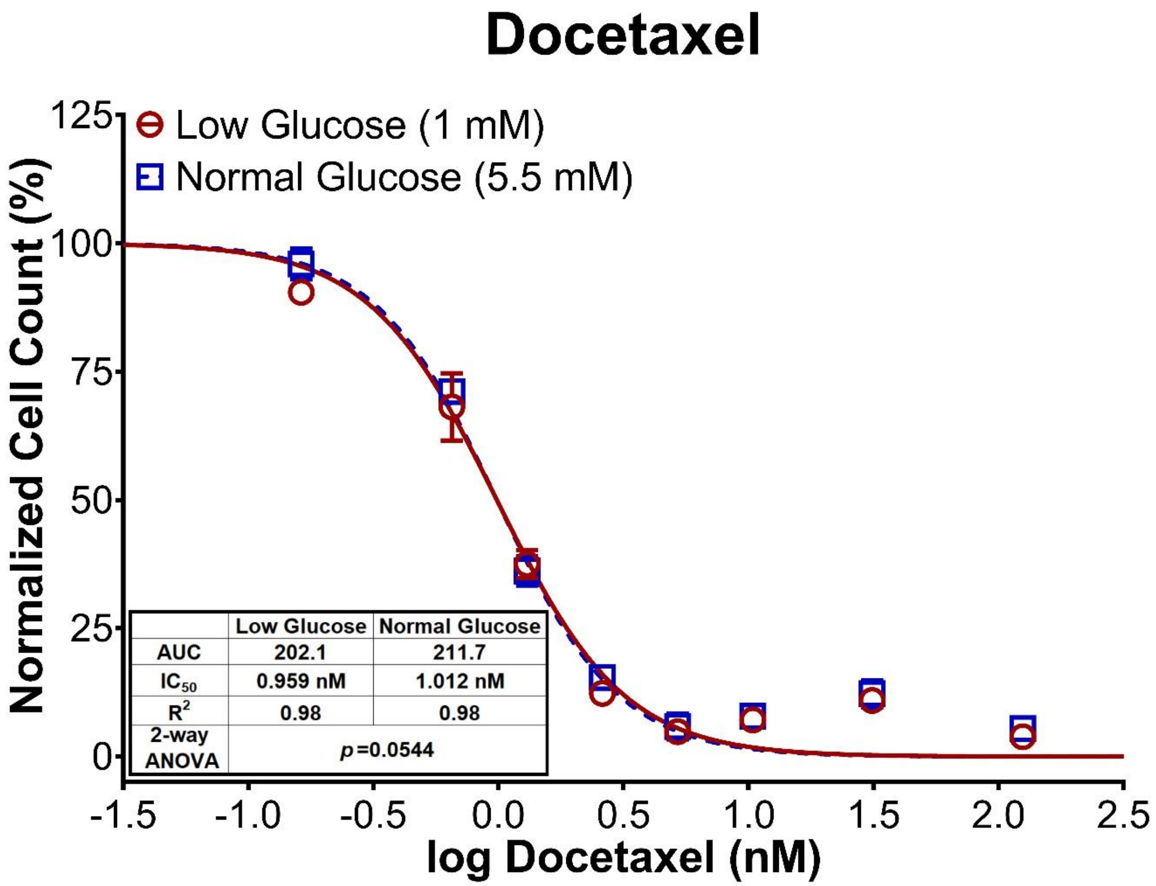

Figure S5. The dose-response curve of PC3 cells after docetaxel treatment for 48 hours in either normal glucose or low glucose media. 4 replicates per group and the experiments were repeated 2 times**.** The statistical analysis was performed by two-way ANOVA.

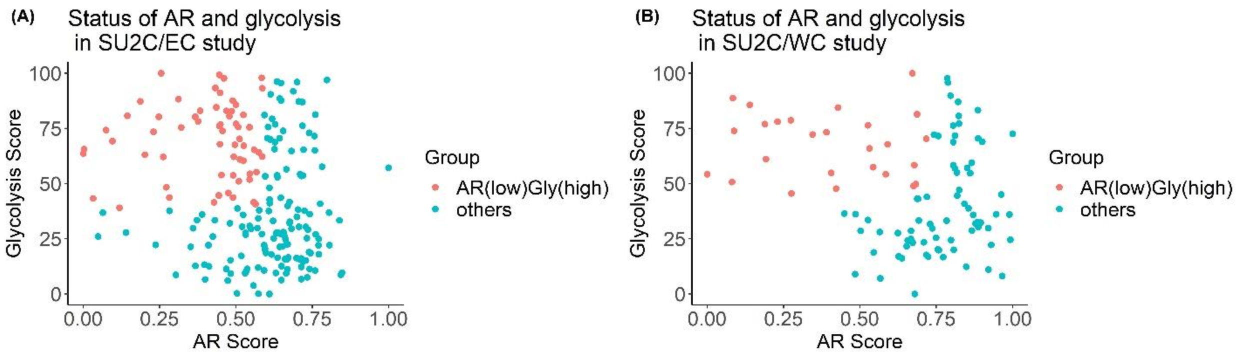

Figure S6. Distribution plots of patients based on AR and glycolysis scores. (A) SU2C/EC study (B) SU2C/WC study. The median is used as a cutoff to classify the low and high groups. Abbreviations: AR: androgen receptor activity; Gly: glycolysis activity; SU2C/EC: Standard Up to Cancer East Coast; SU2C/WC: Standard Up to Cancer West Coast.

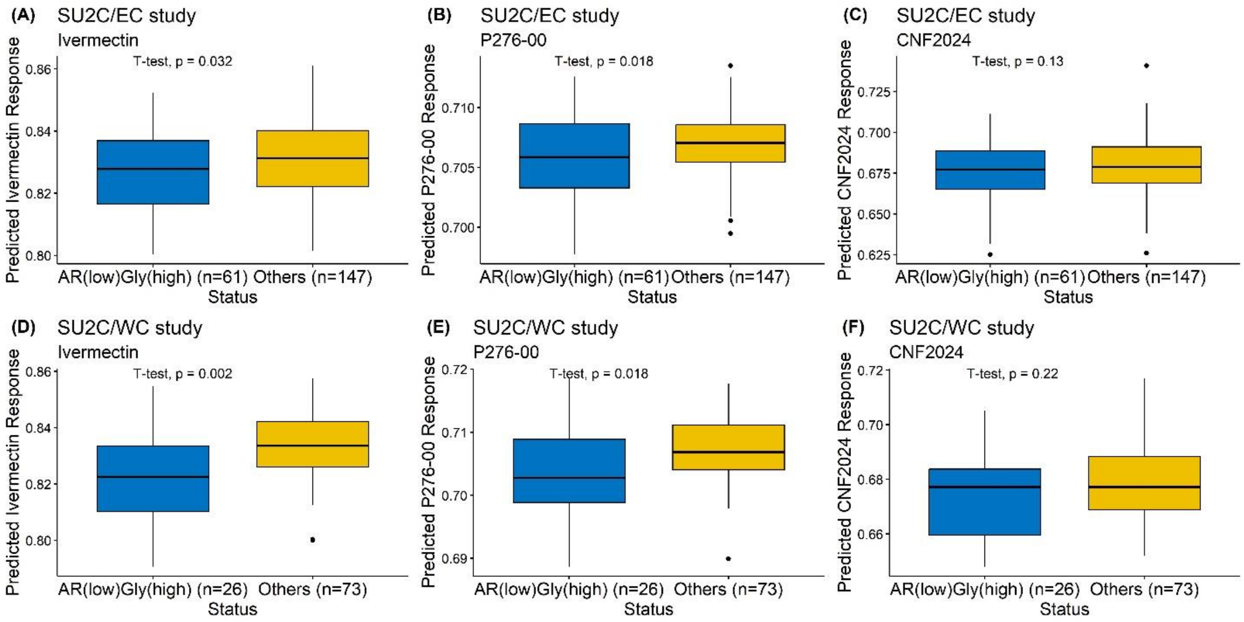

Figure S7. Comparison of predicted drug responses between AR(low) and glycolysis(high) group and the others group**.** (A-C) Comparison of predicted Ivermectin, P276-00, and CNF2024 responses in the SU2C/EC study. (D-F) Comparison of predicted Ivermectin, P276-00, and CNF2024 responses in the SU2C/WC study. The statistical analysis was performed by Welch's t-test. Abbreviations: AR: androgen receptor activity; Gly: glycolysis activity; SU2C/EC: Standard Up to Cancer East Coast; SU2C/WC: Standard Up to Cancer West Coast.

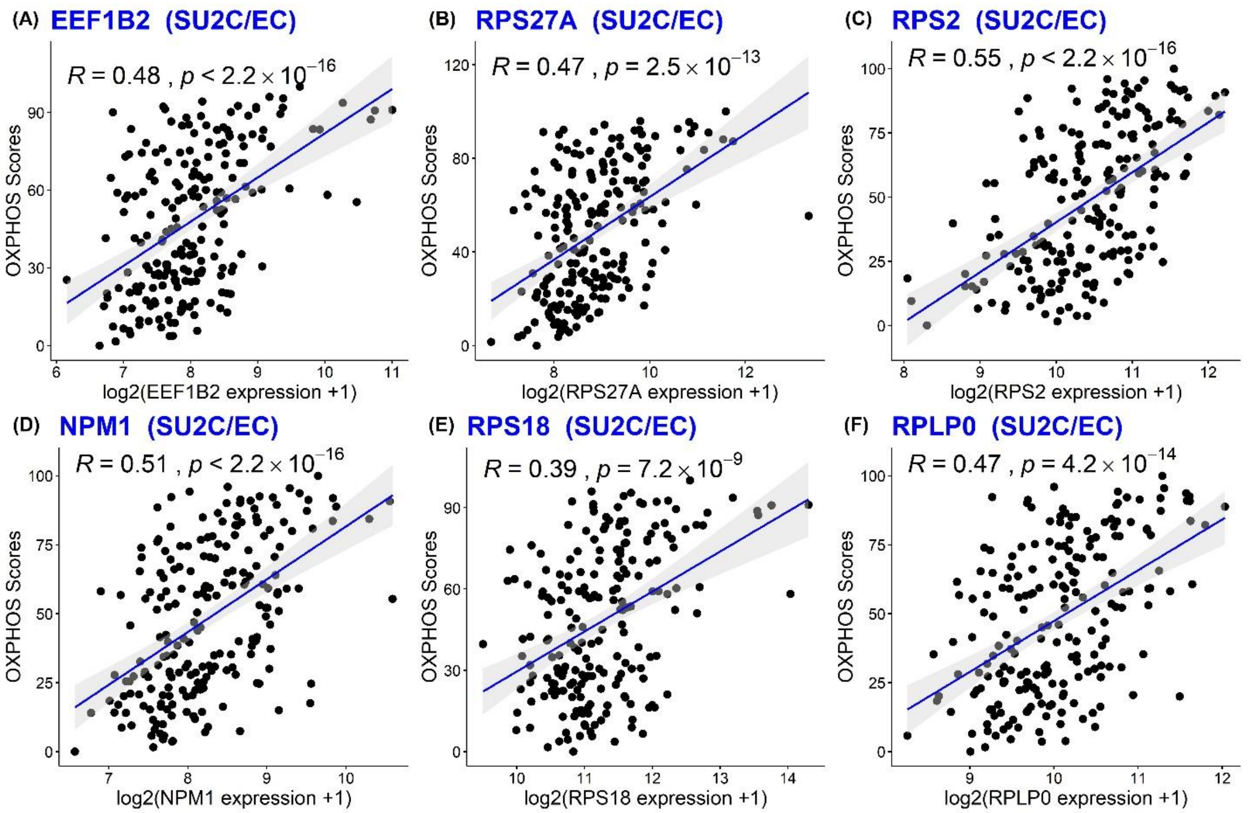

Figure S8. The correlation between gene expression level against OXPHOS score of patients with mCRPC in the SU2C/EC clinical study. (A) *EEF1B2* (B) *RPS27A* (C) *RPS2* (D) *NPM1* (E) *RPS18* and (F) *RPLP0*. For each correlation plot, the Spearman's rho values and its p-value are given. The statistical analysis was performed by Spearman rank correlation and Student's t-test with a significance level α=0.05. Abbreviations: SU2C/EC: Standard Up to Cancer East Coast; SU2C/WC: Standard Up to Cancer West Coast; OXPHOS: oxidative phosphorylation.

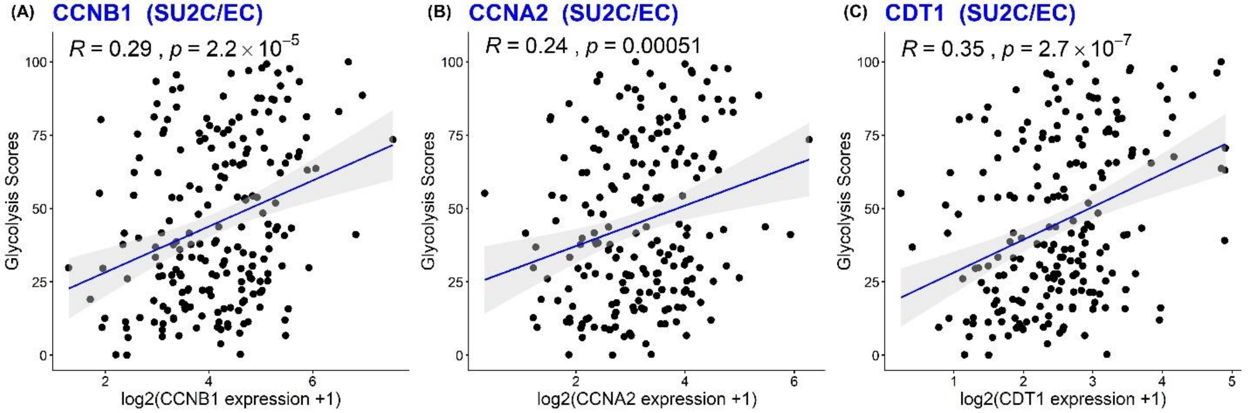

Figure S9. The correlation between gene expression level against glycolysis score of patients with mCRPC in the SU2C/EC clinical study. (A) C*CNB1* (B) *CCNA2* (C) *CDT1.* For each correlation plot, the Spearman's rho values and its p-value are given. The statistical analysis was performed by Spearman rank correlation and Student's t-test with a significance level

$\alpha=0.05$. Abbreviations: SU2C/EC: Standard Up to Cancer East Coast; SU2C/WC: Standard Up to Cancer West Coast.
