# Supplementary material for: Computational Modeling to Identify Drugs Targeting Metastatic Castration-Resistant Prostate Cancer Characterized by Heightened Glycolysis
Source: Pharmaceuticals (Basel). 2024 Apr 29;17(5):569. doi: 10.3390/ph17050569 (PMC11124089; doi:10.3390/ph17050569)
Supplement: Supplementary file 1 [file pharmaceuticals-17-00569-s001.zip › Supplementary Table S1.pdf]

Computational modeling to identify drugs targeting metastatic castration-resistant prostate cancer characterized by heightened glycolysis

Authors: Mei-Chi Su[1], Adam M. Lee[1], Weijie Zhang[2], Danielle Maeser[2], Robert F. Gruener[1], Yibin Deng[3], R. Stephanie Huang[1,2]*

Affiliations:

1. Department of Experimental and Clinical Pharmacology, College of Pharmacy, University of Minnesota, Minneapolis, MN 55455, USA
2. Bioinformatics and Computational Biology, University of Minnesota, Minneapolis, MN 55455, USA
3. Department of Urology, Masonic Cancer Center, University of Minnesota Medical School, Minneapolis, Minnesota, USA

Addresses for Correspondence:

R. Stephanie Huang, PhD
Experimental and Clinical Pharmacology
University of Minnesota College of Pharmacy
B-138 Phillips-Wangensteen Building
Minneapolis, MN 55455, United States
Phone: 612-625-1372
Email: rshuang@umn.edu

Sources of Funding:

RSH received funding from NIH/NCI Grants R01CA204856, R01CA229618 and NCI Contract No. 75N91019D00024, Task Order No. 75N91020F00003. She also received funding from the University of Minnesota (UMN) OACA Faculty Research Development grant, a GIA award, a SURRGE award from the College of Pharmacy and a Masonic Cancer Center CRTI Exceptional Translational Research award.

Disclosures: The authors have no other relevant affiliations or financial involvement with any organization or entity with a financial interest in or financial conflict with the subject matter or materials discussed in the manuscript.

Conflict of Interest Statement: The authors declare no conflicts of interest.

Table S1: Finalized preliminary drug candidates list.

Name	Phase	MOA	category	high_thoughtput_screening
**AZD5438**	Phase 1	CDK inhibitor	Drugs for high glycolysis	PRISM
**Birinapant**	Phase 2	XIAP inhibitor	Dual effect drugs	PRISM
**CNF-2024**	Phase 2	HSP inhibitor	Dual effect drugs	PRISM
**Canertinib**	Phase 3	EGFR inhibitor	Drugs for high glycolysis	PRISM
**Carmofur**	Launched	thymidylate synthase inhibitor	Dual effect drugs	PRISM
**DMH1**	Preclinical	ALK tyrosine kinase receptor inhibitor	Dual effect drugs	PRISM
**Darapladib**	Phase 3	phospholipase inhibitor	Dual effect drugs	PRISM
**Eltrombopag**	Launched	thrombopoietin receptor agonist	Dual effect drugs	PRISM
**Exatecan-mesylate**	Phase 3	topoisomerase inhibitor	Drugs for high glycolysis	PRISM
**FR-122047**	Preclinical	cyclooxygenase inhibitor	Dual effect drugs	PRISM
**Fenoprofen**	Launched	prostaglandin inhibitor	Dual effect drugs	PRISM
**GSK1070916**	Phase 1	Aurora kinase inhibitor	Drugs for high glycolysis	PRISM
**GSK2141795**	Phase 2	AKT inhibitor	Dual effect drugs	PRISM
**Ganetespib**	Phase 3	HSP inhibitor	Drugs for high glycolysis	PRISM
**HBX-41108**	probe	inhibitor of the deubiquitinase activity of USP7	Drugs for high glycolysis	CTRP
**Indisulam**	Phase 2	CDK inhibitor	Drugs for high glycolysis	PRISM
**Indisulam**	Phase 2	CDK inhibitor	Dual effect drugs	PRISM
**Ivermectin**	Launched	benzodiazepine receptor agonist	Dual effect drugs	PRISM
**Nilotinib**	Launched	Abl kinase inhibitor, Bcr-Abl kinase inhibitor	Dual effect drugs	PRISM
**P276-00**	Phase 2	CDK inhibitor	Drugs for high glycolysis	PRISM
**P276-00**	Phase 2	CDK inhibitor	Dual effect drugs	PRISM
**Papaverine-Hydrochloride**	Phase 2	phosphodiesterases inhibitor and calcium channel inhibitor	Dual effect drugs	PRISM
**Platin**	FDA	DNA alkylator; organoplatinum reagent	Drugs for high glycolysis	CTRP

**RS-67506**	Preclinical	serotonin receptor partial agonist	Dual effect drugs	PRISM
**Rapamycin**	Launched	mTOR inhibitor	Dual effect drugs	PRISM
**SB-431542**	probe	inhibitor of the transforming growth factor beta type 1 receptor	Drugs for high glycolysis	CTRP
**SNS-314**	Phase 1	Aurora kinase inhibitor	Drugs for high glycolysis	PRISM
**SNS-314**	Phase 1	Aurora kinase inhibitor	Dual effect drugs	PRISM
**TU-2100**	Phase 2	anti-acne prodrug	Dual effect drugs	PRISM
**Torin-2**	Preclinical	mTOR inhibitor	Drugs for high glycolysis	PRISM
**Torin-2**	Preclinical	mTOR inhibitor	Dual effect drugs	PRISM
**linifanib**	clinical	inhibitor of VEGFRs	Drugs for high glycolysis	CTRP
